# The Relationship Between Anxiety, Depressive Symptoms, and Psychological Crisis in College Students: A Longitudinal Network Analysis

**DOI:** 10.3390/bs16071136

**Published:** 2026-07-07

**Authors:** Yuying Tong, Xuemei Li, Jing Yin

**Affiliations:** 1 College of Innovation and Entrepreneurship Education, Heilongjiang University, Harbin 150000, China; lxm242706@163.com; 2 Centre for Mental Health Education, Heilongjiang University, Harbin 150000, China

**Keywords:** anxiety, depression, psychological crisis, longitudinal network analysis

## Abstract

A six-month follow-up study with two waves was conducted among 10,170 college students. Longitudinal network analysis was used to examine the core symptoms of anxiety and depression as well as the manifestation of psychological crisis; to explore the network relationships among anxiety, depression, and psychological crisis; and to test the temporal stability of these network relationships. The results are as follows: (1) The core symptoms of anxiety and depression among college students were somatic symptoms and affective disturbance at T1, and cognitive symptoms and affective disturbance at T2; the core node of psychological crisis at both T1 and T2 was social support deficit and self-cognitive distress. (2) Psychomotor disturbance was the strongest predictor of other nodes. (3) The overall network structure from T1 to T2 remained relatively stable. These findings reveal the relationship between anxiety/depressive symptoms and psychological crisis among college students, providing a new perspective for psychological crisis intervention in this population.

## 1. Introduction

College students are at a critical turning point in their psychological development, facing multiple concurrent challenges, such as academic pressure, employment competition, and identity reconstruction. Regarding the prevalence of mental health problems, meta-analyses show that the detection rate of anxiety among college students is 13.7% and that of depression is 20.8% (Y. M. Chen et al., 2022). Employment pressure has become a key factor affecting college students’ mental health (Xie, 2022). The “involution” triggered by excessive competition further exacerbates students’ psychological burden (Ma, 2025), and the superposition of multiple pressures makes college students’ psychological problems increasingly prominent, particularly suicidal ideation, which is rising in this population (Huang et al., 2022). Psychological crisis is closely linked to self-injury and suicide (Olga et al., 2022), highlighting the urgency of exploring its underlying mechanisms. Psychological crisis refers to an emergency state in which individuals, confronted with sudden, major, or persistent events, are unable to utilize their existing cognitive and coping strategies to maintain or restore their psychological equilibrium, resulting in acute emotional, cognitive, or behavioral dysfunction that may even endanger their or others’ safety (Isabel et al., 2021). Among college students, psychological crisis is multidimensional, generally encompassing repetitiveness, destructiveness, complexity, and potential positivity (Peng & Han, 2023). Its triggering mechanisms are intricate, often resulting from the interplay of multiple factors such as personality, family, and social environment. Particularly among college students, the interactions of multiple social and psychological factors, including self-esteem (Sun et al., 2024), employment pressure (Y. Chen & Li, 2025), parenting style (Gao et al., 2023), perceived discrimination (Xu & Li, 2025), and lack of sense of meaning in life (Olga et al., 2022), often serve as variables that disrupt psychological equilibrium. It should be noted that the psychological crisis of college students examined in this study falls within the scope of situational and developmental psychological distress—that is, acute emotional, cognitive, or behavioral dysfunction that occurs when individuals face developmental challenges such as academic pressure, employment competition, and interpersonal relationships, and their coping resources are insufficient to maintain psychological equilibrium. This type of psychological distress is fundamentally different in nature, course, and intervention strategies from major mental disorders (e.g., schizophrenia, bipolar disorder, and other conditions requiring long-term medication). The college students involved in this study are all from non-clinical samples, and their psychological crisis primarily manifests as anxiety and depressive symptoms and self-regulation dysfunction triggered by adjustment problems, rather than as severe psychopathological states.

Anxiety and depression are major antecedents and key risk factors for psychological crisis (Zeng et al., 2024); 72% of crisis events are associated with comorbid anxiety and depression, and suicidal ideation is 3.2 times likelier in individuals with comorbidities than in those with a single symptom (Peng & Han, 2023). As key risk factors, anxiety and depression can significantly predict self-injurious and suicidal behaviors (Wei et al., 2022; Yin et al., 2024). College students in a chronic state of anxiety experience continuous depletion of psychological energy and physiological resources, gradually falling into a predicament of physical and mental exhaustion. Once they encounter a sudden stressful event and lack effective coping strategies, maladaptive anxiety can rapidly trigger a psychological crisis, manifesting as emotional collapse and behavioral dyscontrol (Papenfuss et al., 2025). Meanwhile, college students immersed in a prolonged depressive state characterized by a vicious cycle of “low mood–negative cognition–social detachment” eventually distort death into the only way out, thereby inducing extreme crisis behaviors such as self-injury or suicide (M. T. Li et al., 2023). Anxiety and depression do not exist independently; rather, through a complex dynamic interaction network, they gradually erode individuals’ psychological resilience and ultimately breach the “psychological crisis threshold,” manifesting as academic interruption, self-injurious behavior, or suicidal attempts (Isabel et al., 2021; Peng & Han, 2023). Therefore, effective intervention and management of anxiety and depression constitute an important line of defense in preventing psychological crisis. Notably, the detection rate of emotional disorders such as anxiety and depression among college students has been increasing year by year (X. Han et al., 2025), indicating an increase in the risk of psychological crisis in this group. This highlights the urgent need to construct a psychological crisis early warning system centered on emotional health monitoring.

Watson proposed the two-factor model of emotion, with positive and negative affects as two relatively independent dimensions (Watson & Tellegen, 1985). Anxiety and depression mainly reflect unipolar negative affect (W. Liu et al., 2018), and psychological crisis also triggers negative affect (Qi, 2024). According to the cognitive information processing theory model, an individual’s cognitive appraisal process of external information directly determines the nature of emotional responses (T. T. Li et al., 2021); stressful events activate the individual’s emotional response system, in which anxiety and depression, as typical forms of negative affect, follow the transmission pathway of “stress–cognitive appraisal–emotional response.” When individuals adopt cognitive reappraisal strategies, the risk of psychological crisis can be effectively reduced (Sun et al., 2025). There is a direct logical correspondence between the above theoretical framework and the network analysis approach, which also serves as the methodological rationale for choosing network analysis over traditional regression analysis in this study. First, the two-factor model of emotion provides a theoretical basis for the selection and grouping of nodes in network analysis—namely, that anxiety, depression, and psychological crisis can all be viewed as manifestations of different sets of nodes within the negative affect spectrum, rather than as completely independent disease entities. Network analysis allows researchers to reveal the co-occurrence structure and relative importance among symptoms by estimating partial correlation coefficients between nodes, without making a priori causal assumptions about the variables (C. Chen et al., 2021). Second, cognitive information processing theory emphasizes the transmission pathway of “stress–cognitive appraisal–emotional response,” which provides theoretical guidance for the expected direction of edges in the network: that is, cognitive appraisal nodes (e.g., cognitive symptoms) should be strongly connected to emotional response nodes (e.g., affective disturbance), and this connection is likely to exhibit cross-temporal stability. Finally, emotion regulation framework theory suggests that differences in individuals’ coping strategies when facing stress affect the nature and intensity of emotional responses, which provides a theoretical explanation for understanding differences in node centrality within the network—namely, that nodes occupying central positions in the network may be key targets of emotion regulation failure (C. Chen et al., 2021). Based on the aforementioned theoretical–methodological correspondence, the present study adopted network analysis, aiming to identify core symptom nodes within each dimension of anxiety, depression, and psychological crisis; to examine the connection patterns among dimensions; and to test their cross-temporal stability and predictive relationships, all under the guidance of theories of emotion and cognition.

Studies on psychological crisis have mostly focused on identifying influencing factors and designing intervention strategies, yet they generally neglect an in-depth analysis of the relationship between the intrinsic dimensions of anxiety and depression and psychological crisis. Most studies involve cross-sectional correlational analysis of factors, failing to reveal the dynamic evolutionary mechanisms of symptoms. They have not, from the perspective of the dimensions of anxiety and depression themselves, deeply analyzed the relationship between these dimensions and psychological crisis, nor have they clarified which specific dimensions of anxiety and depression have stronger predictive effects on psychological crisis. Often, anxiety and depression are bundled together due to some similar symptoms when studying their impact on psychological crisis. Network analysis can clarify the intrinsic relationships among depressive symptoms, anxiety symptoms, and psychological crisis, identify their comorbidity, and explore nodes at the central positions of the network that occupy a dominant role, thereby helping to reveal the internal structure of psychological constructs and leading to various indirect inferences (Briganti et al., 2018; Dalege et al., 2016). For example, factors that influence a particular dimension of anxiety or depression may have similar effects on psychological crisis, thus providing a more detailed theoretical basis for intervention pathways.

Based on the above theoretical framework and gaps in the literature, in the present study, we propose the following four research questions:

**Q1:** At the two time points T1 and T2, what are the core symptom nodes within each of the three dimensions—anxiety, depression, and psychological crisis—among college students, and do these core nodes exhibit cross-temporal stability between the two time points?

**Q2:** What is the edge structure of the network among the three dimensions of anxiety, depression, and psychological crisis, and which dimensional pairs show the strongest connections?

**Q3:** Do symptom nodes at T1 have prospective predictive associations with psychological crisis nodes at T2 over time, and which nodes have the strongest out-expected influence in the longitudinal network?

**Q4:** Do the centrality indices of both the cross-sectional and longitudinal networks demonstrate good stability (CS-coefficient > 0.5)?

Addressing these four research questions, this study adopts a longitudinal network analysis approach. By tracking the temporal evolution of the symptom networks of anxiety and depression among college students, we systematically examine how different dimensions of anxiety and depression evolve over time and influence the occurrence and development of psychological crisis, with the aim of providing a theoretical basis for its early identification and precise intervention.

## 2. Materials and Methods

### 2.1. Participants

In this study, we employed a two-wave longitudinal design with a six-month interval between waves. A cluster sampling method was adopted. First, three universities in Heilongjiang Province were purposively selected to represent comprehensive, normal, and science and engineering institutions, respectively. Within each selected university, classes or majors served as the primary sampling clusters, and all students in the selected clusters were invited to participate.

The initial sample size was determined based on two considerations: (a) the expected detection rates of anxiety and depression (approximately 11.8% and 19.3%, respectively, according to prior pilot data), and (b) the requirement for parameter stability in the planned longitudinal network analysis. To account for an estimated attrition rate of approximately 50% over the six-month follow-up period, we set a relatively large baseline sample. The sample size was allocated proportionally across the three universities according to each institution’s total registered student population to ensure adequate representativeness. Ultimately, a total of 21,358 students were enrolled at the first wave (T1, March 2022). After excluding participants lost to follow-up and new additions at the second wave (T2, September 2022), a valid sample of 10,170 participants was retained for the primary analyses.

Inclusion criteria were: (a) full-time undergraduate or postgraduate students at the three selected universities; and (b) provision of valid informed consent. Exclusion criteria were: (a) severe physical illness or diagnosed mental disorders that prevented independent completion of the questionnaires; and (b) missing data at either T1 or T2.

Given that data collection occurred during the COVID-19 prevention and control period, all surveys were administered online via a secure electronic platform. Trained research assistants (coordinated by the mental health counseling center of each university) distributed questionnaire links through class counselors and student group chat applications. Data collection was completed by these research assistants under the supervision of the corresponding author’s institution.

Prior to the survey, we received ethical approval from the Ethics Committee of the corresponding author’s institution. Informed consent was obtained electronically on the first page of the questionnaire; the consent form detailed the study’s purpose, the principle of voluntary participation, strict data confidentiality measures, and the participants’ right to withdraw from the survey at any time without penalty. To minimize potential psychological harm, we provided participants with contact information for the university’s mental health services and a 24 h psychological crisis hotline at both the beginning and end of each survey. Additionally, for participants whose scores indicated high psychological risk, the system automatically triggered referral prompts. Specifically, the questionnaire system incorporated a tiered risk assessment algorithm: when a participant’s score on the anxiety, depression, or psychological crisis scales exceeded the pre-established clinical cutoff, the system automatically generated a risk alert. For those at moderate risk (i.e., scores exceeding the cutoff but not meeting emergency intervention criteria), research assistants proactively contacted the participant within 24 h via the provided contact information, conducted a brief psychological status assessment, and guided them to make an appointment at the university counseling center. For those at high risk (i.e., scores exceeding the cutoff and concurrently reporting self-injury or suicidal ideation), the system immediately sent an emergency notification to the research assistants and the on-duty staff at the university counseling center. Professional staff conducted telephone crisis intervention within 1 h and initiated face-to-face referrals based on the assessment results. All risk alerts and intervention records were stored separately with encryption and strictly isolated from the survey data. All research assistants received training in crisis identification and basic intervention skills provided by the university counseling centers prior to data collection.

Regarding data anonymization and sharing, all data were rigorously de-identified before export for analysis. Specific measures included: (1) the removal of all direct identifiers, including names, student identification numbers, national ID numbers, contact phone numbers, and email addresses; (2) the generalization of indirect identifiers (e.g., college, major, and grade) by, for example, consolidating specific majors into broader disciplinary categories and replacing exact ages with age ranges; and (3) the assignment of a unique study code to each participant. The correspondence table linking raw data to the codes was stored separately with encryption on the internal server of the corresponding author’s institution and was accessible only to research team members during the data analysis period. The raw data (containing any identifiable information) will not be shared and will be destroyed within five years after the completion of the study in accordance with institutional regulations. All data processing procedures complied with the Declaration of Helsinki and relevant Chinese data protection regulations.

At T1, among the 21,358 initial respondents, 2515 individuals (11.8%) were identified as having an anxiety tendency and 4123 (19.3%) as having a depression tendency. At T2, among the 10,170 follow-up participants, 1366 (13.4%) had an anxiety tendency and 2254 (22.2%) had a depression tendency. Subsequent longitudinal network analyses were conducted on the above data to examine the core symptoms, temporal stability, and network relationships among anxiety, depression, and psychological crisis.

### 2.2. Measures

#### 2.2.1. Self-Rating Anxiety Scale, SAS

The 20-item SAS (5 reverse-scored and 15 forward-scored items) was used to assess the anxiety status of the study participants. According to the frequency of symptom occurrence, items are rated on a four-point scale ranging from 1 = “none or rarely” to 4 = “most or all of the time.” The raw scores were standardized (raw score × 1.25) before symptom assessment. A standardized score below 50 indicates normal, while a score of ≥50 indicates the presence of anxiety symptoms. The scale comprises four dimensions: cognitive symptoms, somatic symptoms, motor symptoms, and sleep symptoms (Duan & Sheng, 2012). In this study, the scale’s Cronbach’s α was 0.78 at T1 and 0.80 at T2, both indicating good reliability.

#### 2.2.2. Self-Rating Depression Scale, SDS

The SDS was used to assess the depression status of the study participants. It consists of 20 items, of which 10 are reverse-scored and 10 forward-scored, all reflecting subjective feelings of depression. According to the frequency of symptom occurrence, items are rated on a four-point scale ranging from 1 = “none or rarely” to 4 = “most or all of the time.” The raw scores were standardized (raw score × 1.25) before symptom assessment. A standardized score below 53 indicates normal, while a score of ≥53 indicates the presence of depressive symptoms. The scale comprises four dimensions: affective disturbance, somatic disturbance, psychomotor disturbance, and psychological disturbance (Duan & Sheng, 2012). In this study, the scale’s Cronbach’s α was 0.87 at T1 and 0.88 at T2, both indicating good reliability.

#### 2.2.3. Campus Psychological Crisis Scale for College Students

The College Student Campus Psychological Crisis Scale was used to assess the psychological crisis status of the study participants. It consists of 19 items, with no reverse scoring. The manifestations related to psychological crisis were quantified according to the “degree of agreement,” and the “degree of match between the target behavior/state and one’s own situation” was classified into five levels, ranging from 1 = “completely inconsistent” to 5 = “completely consistent.” The scale comprises three dimensions: social support deficit and self-cognitive distress, self-regulation and psychosomatic dysfunction, and negative events and crisis behavior. In this study, the scale’s Cronbach’s α was 0.88 at T1 and 0.89 at T2, both indicating good reliability.

### 2.3. Data Processing

In this study, we used R version 4.5.2 statistical software for network analysis and data visualization. In the network analysis of this study, we selected four centrality indices to evaluate the importance of nodes: strength, closeness, betweenness, and expected influence (EI). Among these, node strength is measured by summing the absolute values of the weights of all connections between the node and its adjacent nodes; the higher the value, the more significant the role of the node in the entire network. Node closeness reflects the proximity of the node to all other nodes in the network, represented by the sum of the reciprocals of the shortest path lengths to all other nodes; the larger this value, the more efficient the node in the transmission of information or influence. Node betweenness characterizes the mediating role of the node in connecting different parts of the network, quantified by calculating the total number of times the node appears on the shortest paths between all other pairs of nodes; the higher the value, the more critical the bridging function of the node in maintaining network connectivity. Node EI refers to the total effect on all nodes in the entire network when a small, sustained intervention is applied to a given node; it builds upon strength by considering the dynamic changes in the entire network, that is, the sum of the direct and indirect effects of the node (Epskamp et al., 2018a).

#### 2.3.1. Correlation Analysis

The psych package was used for the calculation of the correlation coefficient; the ggplot and Corrplot packages were used for data visualization; the reshape2 package was used for data reshaping (e.g., from wide format to long format); the dplyr package was used for data manipulation and transformation.

#### 2.3.2. Regularized Partial Correlation Network Analysis

The EBICglasso function from the psych package was used to compute regularized partial correlations (Epskamp et al., 2018b). EBICglasso combines with the Graphical LASSO in the Gaussian graphical model to select regularization parameters, obtaining more stable and sparse partial correlation estimates, thereby better revealing conditional independence relationships among variables for easier interpretation. The qgraph and bootnet packages were used for network analysis and visualization of the data, and the bootnet package was used for bootstrap analysis of the network model to assess network stability and obtain centrality indices of each node in the network.

#### 2.3.3. Cross-Lagged Network Analysis

Cross-lagged network analysis combines the cross-lagged panel model and network analysis, aiming to explore the prospective predictive associations among nodes over time. It comprises three basic principles: autoregressive effect, cross-lagged effect, and bidirectional testing effect. The autoregressive effect refers to the predictive effect of a node’s value at T1 on its own value at T2. After controlling for the autoregressive effect of node A at T1, the cross-lagged effect refers to testing whether node B’s value at T1 can predict node A’s value at T2. The bidirectional testing effect refers to simultaneously testing the effects of A → B and B → A and inferring the causal direction by comparing the magnitudes of the effects. In this study, the lm function in R was used to estimate the cross-lagged network model across two time points. The cross-lagged effects were estimated by building linear regression models for each variable at T2 (with all variables at T1 as predictors), and the resulting regression coefficients were organized into a cross-lagged matrix. In addition, we calculated network topological characteristic indices, including network density, connection strength, and the distribution range of edge weights, to quantify the overall structural properties of the cross-lagged network. These indices help to understand the complexity and connection patterns of influence pathways within the variable system, providing a visual and quantitative basis for in-depth exploration of the temporal dynamic interactions among nodes. To enhance the interpretability of the results and create a more intuitive network structure, we used the qgraph package to visualize the cross-lagged matrix as a directed network graph, clearly displaying the time-series predictive relationships among variables through directed edges and curves. The thickness and direction of the edges reflect the strength and direction of the predictive effects, where dark blue edges indicate positive predictive relationships and red edges indicate negative predictive relationships. It should be noted that, although in this study we adopted a longitudinal design, we included only two measurement time points and did not control for all potential confounding variables. Therefore, the predictive associations estimated in the cross-lagged network analysis should not be equated with causal relationships. The out-expected influence reported in this study reflects the statistical predictive power of the severity of a given node at T1 on the severity of other nodes at T2, rather than a direct causal effect. All statements involving “prediction” should be understood as referring to “prospective associations over time” rather than causal inferences (Sorjonen et al., 2025).

#### 2.3.4. Centrality Estimation

The directional nature of the cross-lagged network allows for the calculation of two node centrality indices: out-expected influence (out-EI) and in-expected influence (in-EI). Out-EI refers to the degree to which a node predicts other nodes in the network, whereas in-EI refers to the degree to which a node is predicted by all other nodes in the network. The rowSums and colSums functions were used to calculate these two EI indices. In this study, the definitions and purposes of the centrality estimation indices are as follows: Expected Influence (EI): Used to identify core symptom nodes in the cross-sectional network, that is, nodes that are most strongly connected to other nodes within the same time point. EI takes into account the sign of edge weights and is superior to traditional strength centrality in networks that contain negative associations. Out-Expected Influence (out-EI): Used to identify predictive nodes in the longitudinal cross-lagged network, that is, the prospective predictive power of a node at T1 on other nodes at T2. In-Expected Influence (in-EI): Used to identify nodes that are being predicted in the longitudinal cross-lagged network, that is, the extent to which a node at T2 is predicted by other nodes at T1.

#### 2.3.5. Stability Estimation

The case-dropping bootstrap method was used to test the stability of the network’s centrality indices. The operational principle involves randomly drawing samples of different proportions (range: 50–95%) from the original data, then repeatedly (1000 times in this study) (Dong et al., 2025) re-estimating the network model and calculating the centrality indices under that sample size. Finally, the centrality indices under different sample proportions were correlated with those from the original data. The higher the resulting correlation coefficient, the more stable the centrality indices of the original data; the correlation coefficient obtained in this way is called the centrality stability coefficient (CS coefficient) (Borsboom et al., 2021/2022). In this study, 0.7 was used as the correlation threshold, and it was required that the correlations of 95% of the bootstrap samples were ≥0.7. A CS coefficient of <0.25 indicates poor stability of the centrality indices; 0.25–0.50 indicates good stability; and >0.5 indicates strong stability (Isvoranu et al., 2022).

## 3. Results

### 3.1. Common Method Bias

To assess the potential for common method bias, Harman’s single-factor test was conducted using SPSS version 27.0 (IBM Corp., Armonk, NY, USA). All items of the negative event scale, depression scale, and anxiety scale at T1 and T2 were included separately, and exploratory factor analysis was conducted. The results revealed nine factors with eigenvalues > 1 at T1 and T2, respectively; the variance explained by the first factor was 10.42% and 10.07%, respectively, both below the critical value of 40%, indicating no serious common method bias in this study (Harman et al., 2005).

### 3.2. Correlation Analysis

**Figure 1:**
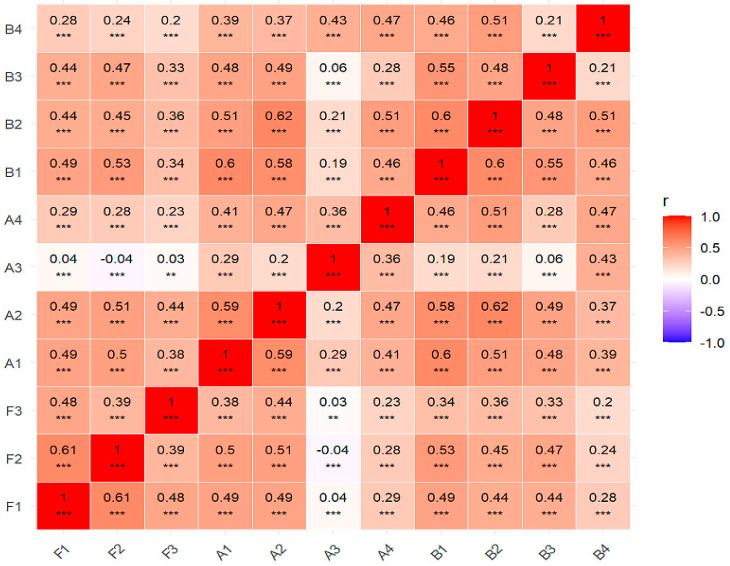
Correlation relationships among dimensions at T1. Note: ** *p* < 0.05 and *** *p* < 0.01.

**Figure 2:**
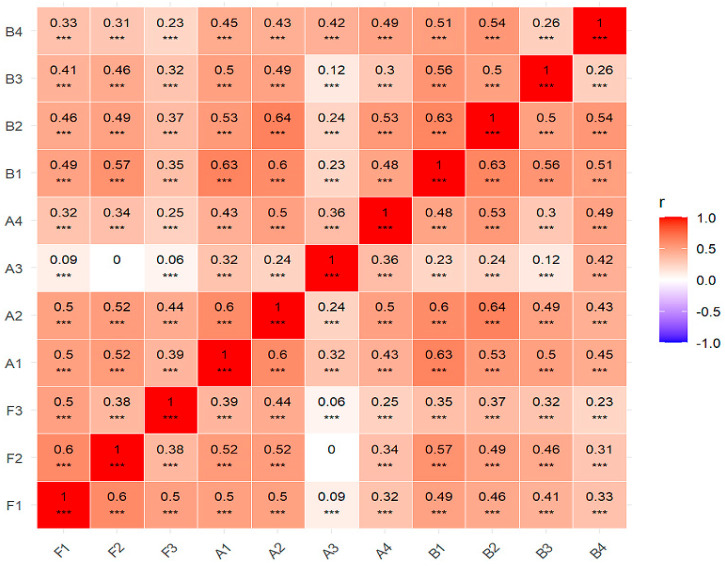
Correlation relationships among dimensions at T2. Note: *** *p* < 0.01.

Correlation analysis was conducted on each dimension of anxiety, depression, and psychological crisis among college students at T1 and T2 (Figure 1 and Figure 2). At T1, the correlations between the three dimensions of psychological crisis and each dimension of anxiety and depression were all significant. At T2, the correlations between F1, F3 and each dimension of anxiety and depression were all significant, while F2 indicated significant correlations with all other dimensions of anxiety and depression except for the motor symptom dimension of anxiety (In this text, all A1 = cognitive symptoms; A2 = somatic symptoms; A3 = motor symptoms; A4 = sleep symptoms; B1 = affective disturbance; B2 = somatic disturbance; B3 = psychomotor disturbance; B4 = psychological disturbance; F1 = social support deficit and self-cognitive distress; F2 = self-regulation and psychosomatic dysfunction; F3 = negative events and crisis behavior) (see Figure 1 and Figure 2).

### 3.3. Regularized Partial Correlation Analysis of the Cross-Sectional Network

**Figure 3:**
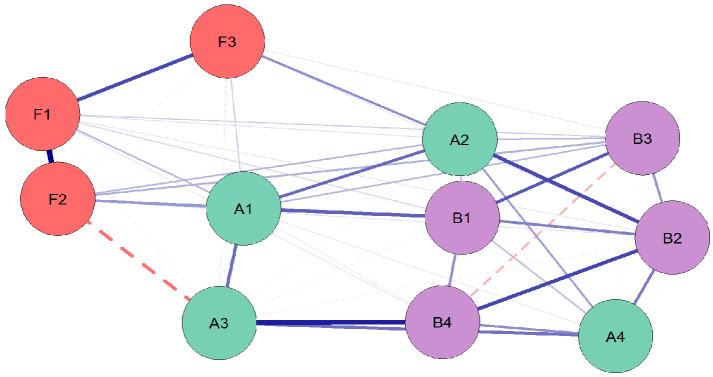
Network structure of college students’ campus psychological crisis, anxiety, and depression at T1. Note: Solid lines indicate positive correlations, and dashed lines indicate negative correlations. Line thickness represents the strength of the association (thicker lines indicate stronger relationships).

**Figure 4:**
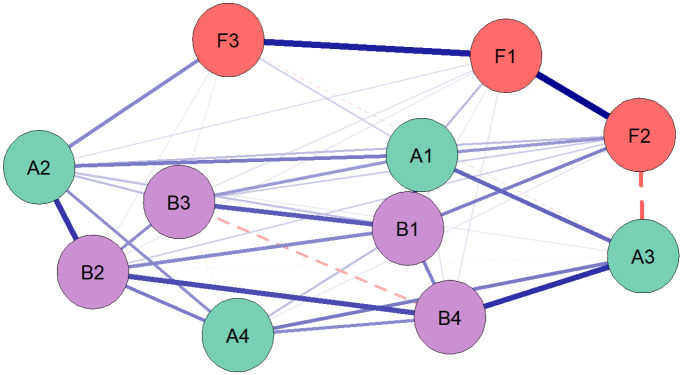
Network structure of college students’ campus psychological crisis, anxiety, and depression at T2. Note: Solid lines indicate positive correlations, and dashed lines indicate negative correlations. Line thickness represents the strength of the association (thicker lines indicate stronger relationships).

**Figure 5:**
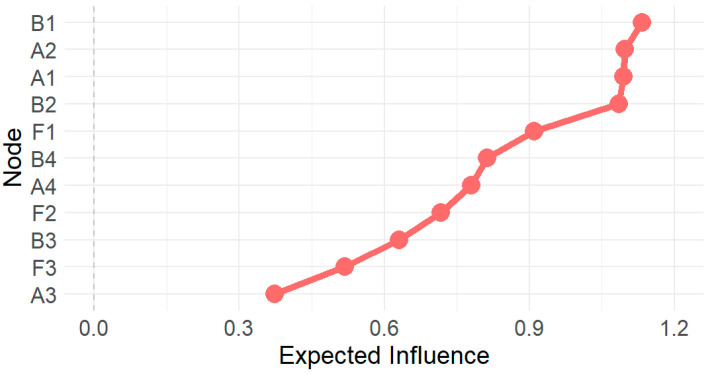
Expected influence of each node at T1.

**Figure 6:**
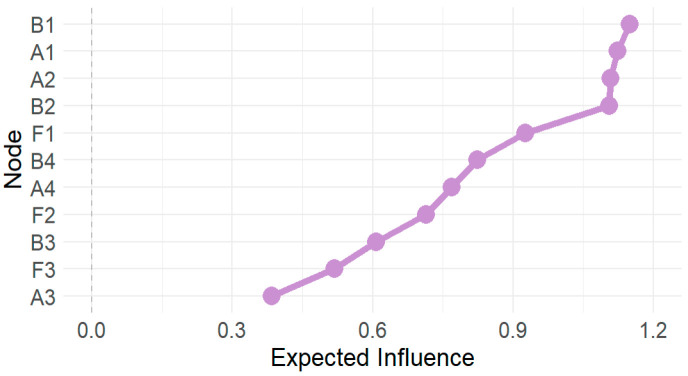
Expected influence of each node at T2.

**Figure 7:**
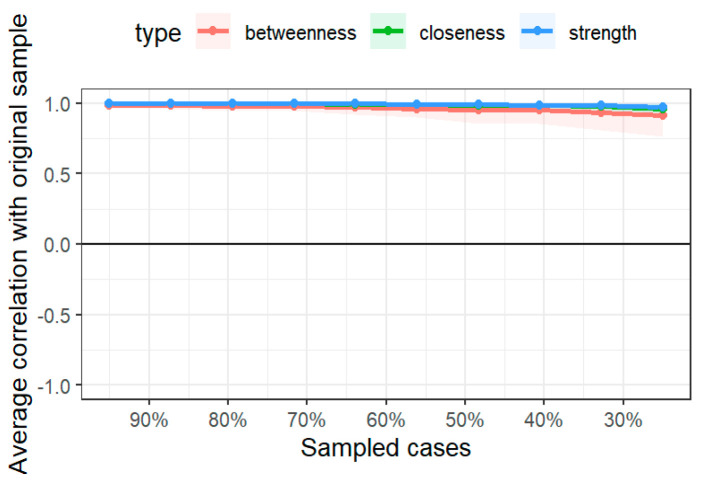
Stability test at T1.

**Figure 8:**
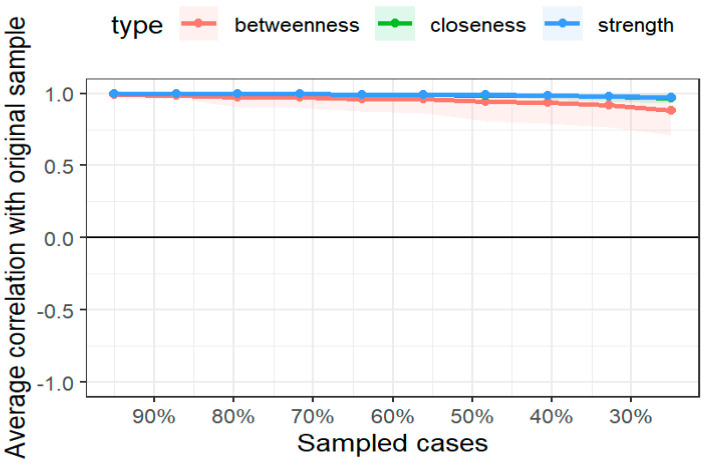
Stability test at T2.

As illustrated in Figure 3 and Figure 4, to evaluate the network structure of anxiety, depression, and psychological crisis at T1 and T2, regularized network estimation was performed on 11 nodes. The network density obtained at T1 was 0.89, with 49 nonzero weighted edges and an average weight of 0.11 (edge coefficients are presented in Appendix A Table A1). The three strongest nodes were somatic disturbance (1.15), affective disturbance (1.14), and F2 (1.10) (Appendix A Table A2). The three strongest edges were: F1–F2 (weight = 0.34), motor symptoms–psychological disturbance (weight = 0.30), and somatic disturbance–psychological disturbance (weight = 0.25). The network density obtained at T2 was 0.91, with 50 nonzero weighted edges and an average weight of 0.11 (edge coefficients are presented in Appendix A Table A3). The three strongest nodes were sleep symptoms (1.19), F2 (1.13), and cognitive symptoms (1.12) (Appendix A Table A2). The three strongest edges were: F1–F2 (0.34), F1–F3 (0.29), and motor symptoms–psychological disturbance (0.27). Overall, the connections between anxiety, depression, and psychological crisis were relatively close at both T1 and T2. Based on the centrality measurement and centrality difference test results (as shown in Figure 5 and Figure 6), the nodes with the highest expected influence (EI) for anxiety, depression, and psychological crisis at T1 were somatic symptoms (EI = 1.10), affective disturbance (EI = 1.13), and F1 (EI = 0.91), respectively. At T2, the corresponding nodes were cognitive symptoms (EI = 1.12), affective disturbance (EI = 1.15), and F1 (EI = 0.93), respectively. Accordingly, it can be inferred that the core symptoms for college students’ anxiety, depression, and psychological crisis are somatic symptoms and cognitive symptoms (for anxiety, at T1 and T2, respectively), affective disturbance (for depression), and F1 (for psychological crisis), as presented in Appendix A Table A4. For the stability test of the two cross-sectional networks at T1 and T2, the three centrality indices of strength, closeness, and betweenness were used, and the results are presented in Figure 7 and Figure 8. The CS coefficients for all three indices were greater than 0.5 at both T1 and T2, indicating good centrality stability of the entire network (see Figure 3, Figure 4, Figure 5, Figure 6, Figure 7 and Figure 8).

### 3.4. Cross-Lagged Network Estimation

**Figure 9:**
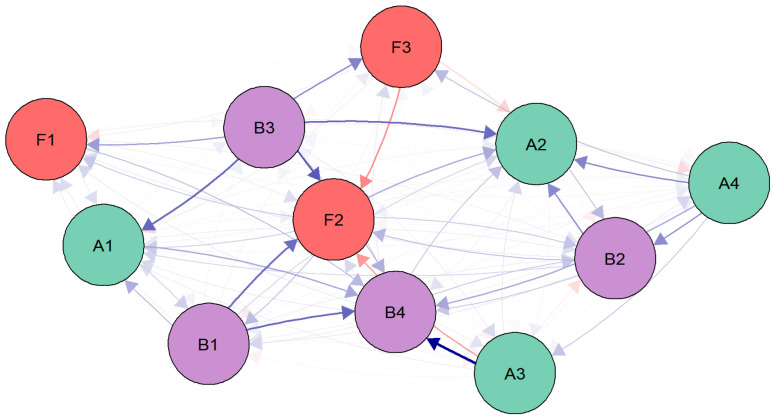
Cross-lagged network diagram from T1 to T2. Blue arrows indicate positive cross-lagged effects, red arrows indicate negative effects. Line thickness is proportional to the absolute regression coefficient. All edges are directed from T1 to T2.

The cross-lagged network of anxiety and depressive symptoms and psychological crisis is presented in Figure 9. Eleven nodes connected by 110 directed edges constitute the cross-temporal cross-lagged network model of psychological crisis, including 90 positive edges (dark blue) and 20 negative edges (red). The network density is 1, with edge weights ranging from −0.13 to 0.27 and an average weight of 0.04 (Appendix A Table A5). The cross-lagged network results revealed that in the T1→T2 network, each dimension of anxiety and depressive symptoms could predict the three dimensions of psychological crisis. The three nodes with the strongest predictive effects in the network were: psychomotor disturbance, affective disturbance, and sleep symptoms. Further analysis of the paths in the cross-lagged network revealed that the three strongest cross-lagged edges in the network were: 1. motor symptoms → psychological disturbance (β = 0.27); 2. psychomotor disturbance → F2 (β = 0.18); 3. affective disturbance → psychological disturbance (β = 0.17) (Insert Figure 9).

### 3.5. Centrality Estimation of the Cross-Lagged Network

**Figure 10:**
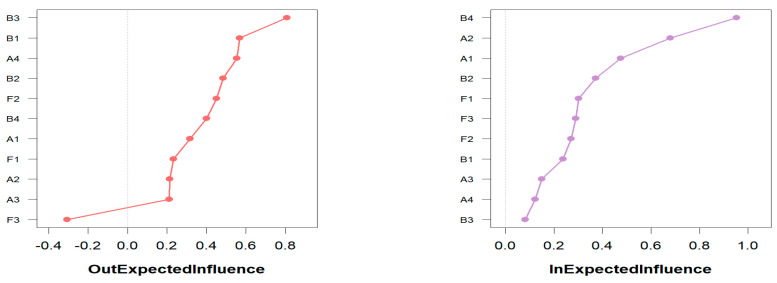
Centrality estimation of the T1 → T2 network.

Centrality estimates are presented in Figure 10. Psychomotor disturbance was the factor with the highest out-EI, followed by affective disturbance, indicating that they have stronger predictive ability for other factors than other nodes. Psychological disturbance was the factor with the highest in-EI, followed by somatic symptoms. In addition, the three dimensions of psychological crisis also had high indices of in-EI, indicating that they are more influenced by other factors in this network (see Appendix A Table A6 for specific values) (see Figure 10).

### 3.6. Stability Analysis of the Cross-Lagged Network

**Figure 11:**
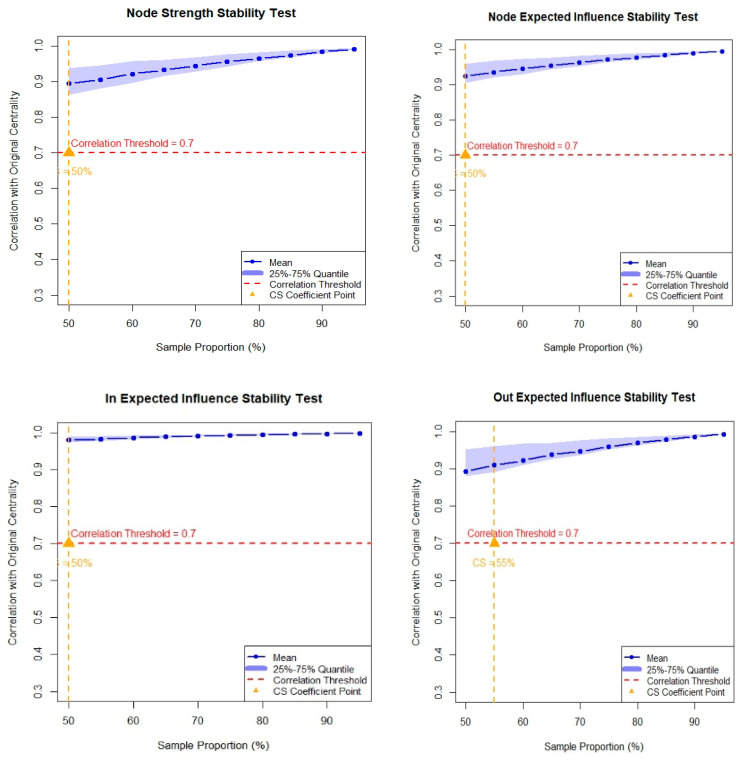
Stability test of the cross-lagged network.

In the T1→T2 network, the case-dropping method was used to test the stability of node strength, (total) node EI, in-EI, and out-EI in the cross-lagged network. The results revealed that the CS coefficients for all centrality indices were greater than 0.5, indicating good centrality stability of the entire network (Figure 11).

## 4. Discussion

Given that anxiety and depression are the most important influencing factors triggering psychological crisis, we explored the dynamic interaction between different dimensions of anxiety, depressive symptoms, and psychological crisis among college students through longitudinal network analysis. Thus, the findings also revealed the predictive relationships among the three across various dimensions, providing an important basis for the intervention of psychological crisis among college students.

### 4.1. Core Components of Anxiety Symptoms, Depressive Symptoms, and Psychological Crisis in College Students

In this study, cross-sectional network analyses were employed to identify the core symptomatic nodes within each of the three dimensions—anxiety, depression, and psychological crisis—at both T1 and T2. The results revealed the dynamic changes and relative stability of the core nodes within each dimension across the two time points.

For the anxiety dimension, the node with the highest expected influence at T1 was somatic symptoms (EI = 1.10), whereas at T2, it shifted to cognitive symptoms (EI = 1.12). This finding indicates that the core symptoms of anxiety among college students underwent a transition from somatic expression to cognitive distortion over the six-month interval, suggesting a dynamic evolution of the anxiety symptom structure. This temporal shift is consistent with previous longitudinal network analyses, which have demonstrated that anxiety symptom networks transition from being driven by somatic fear responses to being dominated by cognitive appraisal symptoms (Blackmore et al., 2026). Ruchkin and Schwab-Stone (Vladislav & Mary, 2014) previously noted that college students experiencing anxiety often present with non-organic physical discomfort, which may serve as an alternative means of communicating emotional distress—essentially a defense mechanism that transforms psychological conflict into somatic symptoms (Vladislav & Mary, 2014). Future interventions should adjust their targets according to the temporal dynamics of the symptom network, with early-stage interventions focusing on somatic symptoms and later-stage interventions shifting toward cognitive symptoms.

Taken together, the core symptom spectrum of depression among college students is characterized by affective disturbance as the central feature, supplemented by somatic disturbance as a secondary component. Accordingly, intervention efforts should prioritize the management of emotional dysregulation while simultaneously addressing somatic discomfort.

For the depression dimension, the core symptom remained consistently stable across both time points, with affective disturbance retaining the highest expected influence at T1 (EI = 1.13) and T2 (EI = 1.15). Moreover, its expected influence value ranked first among all 11 nodes, indicating that affective disturbance is not only the core node within the depression dimension but also exerts the strongest global influence across the entire anxiety–depression–psychological crisis network. When compared with the prior literature, our findings are consistent with those of J. H. Chen et al. (2024), which identified affective disturbance (e.g., self-hatred, irritability, and loneliness) as the core feature of depression in late childhood (J. H. Chen et al., 2024). However, they differ from the pattern observed in high school students, whose depression encompasses both affective disturbance and psychomotor disturbance (e.g., difficulty initiating behaviors) as reported by T. Chen et al. (2025). In our study, the EI values for psychomotor disturbance were relatively low at both time points (T1 = 0.63; T2 = 0.61), suggesting that after entering early adulthood, college students’ psychomotor retardation symptoms may be alleviated to some extent, whereas affective disturbances continue to predominate. In addition, somatic disturbance also exhibited relatively high centrality within the depression dimension (T1 EI = 1.09; T2 EI = 1.11), corroborating the findings of Yiming et al. (2025) and Subaşı et al. (2022), who reported that somatic disturbances such as insomnia, fatigue, and neck pain are commonly observed in depressed patients (Subaşı et al., 2022; Yiming et al., 2025). This also supports the view of Sönmez and Bahçeci (2025) that somatic disturbance impairs college students’ problem-solving abilities, thereby exacerbating cognitive impairment in depression (Sönmez & Bahçeci, 2025). For the psychological crisis dimension, the core node at both time points was identified as “dysregulation of self-regulation and physical–mental functions” (i.e., F1). Cao and Zhang (2025) pointed out that negative self-evaluation—an adverse form of self-cognition—can induce emotional distress, thereby triggering psychological crisis and even undermining self-esteem (Cao & Zhang, 2025). The present study further locates “dysregulation of self-regulation and physical–mental functions” as the initiating node in this causal chain, suggesting that when college students become aware of a “sense of losing control” over their own physical and mental states (e.g., “I cannot control my thoughts;” “my physical reactions exceed my expectations”), they are already in the budding stage of psychological crisis. In contrast to previous perspectives that equate psychological crisis with terminal events such as non-suicidal self-injury or suicidal behavior (Isabel et al., 2021), the findings of this study support viewing psychological crisis as a dynamic developmental continuum within which “dysregulation of self-regulation and physical-mental functions” occupies the foremost position and represents the earliest identifiable and intervenable node. Therefore, in the construction of early warning systems for psychological crisis, items pertaining to “dysregulation of self-regulation and physical–mental functions” should be incorporated into routine screening indicators, rather than waiting until crisis events have already erupted before implementing interventions.

### 4.2. Internal Connection Mechanism Among Anxiety Symptoms, Depressive Symptoms, and Psychological Crisis in College Students

Anxiety and depressive symptoms in college students are inseparable from the emergence of psychological crisis, which is consistent with the findings of Hui et al. (2020). At both T1 and T2, the edge weight of “motor symptoms–psychological disturbance” was relatively high, which not only reflects that these two dimensions are most closely connected and exhibit cross–temporal stability within anxiety and depression, but also indicates that they play a key role in the entire network framework, consistent with previous research (Hodgson et al., 2025). When motor symptoms such as sweating, hand trembling, and inability to concentrate persist, they may coexist empirically with individuals’ feelings of exhaustion and helplessness and show a positive statistical association with depression. Therefore, similar to temperament types, clinically pure anxiety and pure depression are rare; their co-occurrence is more common. Network studies on psychiatric patients have also found that certain symptom clusters of anxiety (panic or fatigue) and depression (emptiness) are closely connected (Zhang et al., 2025). Second, both time points (T1 and T2) indicated that the connections between “social support deficit and self-cognitive distress” and “self-regulation and psychosomatic dysfunction” were stronger than their respective connections with “negative events and negative behaviors.” Thus, the mutual influences between social support and self-cognition, and between self-regulation and psychosomatic function, are stronger than the mutual influence with negative events. It is noteworthy that at both T1 and T2, the connection strength between social support deficit and self-cognitive distress (F1) and self-regulation and psychosomatic dysfunction (F2) was higher than the connections of either dimension with negative events and crisis behavior (F3). This pattern can be tentatively interpreted from the perspective of the Conservation of Resources (COR) theory (Hobfoll, 1989). According to COR theory, social support can be viewed as a conditional resource, and self-regulation as a personality trait resource; both belong to the individual’s core resource cluster, whereas negative events and negative behaviors are resource loss stressors. COR theory posits that resources within the core resource cluster are tightly interconnected, such that when one core resource is depleted, other core resources also tend to suffer. The network analysis results of this study are consistent with this theoretical expectation in terms of the pattern—namely, that the connection strength among core resources is higher than that between core resources and external stressors. It should be emphasized, however, that this theoretical interpretation is a post hoc inference drawn from the network’s structural pattern, rather than a direct test of causal relationships. In addition, at T1 and T2, most dimensions of anxiety and depression were connected to dimensions of psychological crisis; this suggests that the former is statistically closely associated with the latter, and factors that lead to anxiety or depression may be variables that enhance or alleviate psychological crisis. For psychological crisis, especially sudden psychological crisis, its occurrence and development are closely related to an individual’s coping style. From the perspective of the cognitive appraisal theory of stress proposed by psychologists Lazarus and Folkman in 1984, when individuals encounter stressful events, they determine their coping styles through ‘primary appraisal’ (judging whether the event constitutes a threat) and ‘secondary appraisal’ (evaluating their own coping resources and strategies). This theoretical framework provides a possible explanation for the pattern observed in this study—namely, that most dimensions of anxiety and depression are connected to dimensions of psychological crisis. Specifically, when individuals adopt inappropriate coping styles (e.g., excessive avoidance, emotional suppression, or negative attribution), their anxiety and depressive symptoms may be exacerbated, and these emotions themselves may further interfere with their ability to cope effectively (Jiang & Wang, 2022). However, this explanation awaits further scrutiny and validation through more refined experimental designs in future research (Jiang & Wang, 2022). From a cross-sectional perspective, in this process, anxiety and depression can be either emotional reactions triggered by psychological crisis or psychological motives that cause psychological crisis to persist or even intensify.

### 4.3. Relationship Between Anxiety Symptoms, Depressive Symptoms, and Psychological Crisis in College Students

The cross-lagged network analysis of anxiety, depressive symptoms, and psychological crisis among college students revealed that psychomotor disturbance had the largest out-expected influence in the network, that is, the highest degree of statistical prediction of other nodes, suggesting that psychomotor disturbance may be the most predictive node in the entire network. However, it should be clarified that “predictive power” here refers to prospective statistical associations over time, rather than a core factor in a causal sense. According to the emotional framework theory, for psychological crisis among college students, emotional regulation is determined using multifactor interactions and varies by person, situation, and strategy. In the future, we should focus on emotional states, such as depression, as a key point for prevention and intervention. First, depression is closely related to family structure and parenting style. The direct resource for college students to obtain social support is the family. When encountering psychological distress, a supportive family can help them get through it better; otherwise, it can trigger depressive emotions and thus lead to a psychological crisis (Ye et al., 2024). From a developmental psychology perspective, college students’ emotional attachment figures gradually expand from parents to peers, making peers an important source of their social support. Research has shown that peer support exerts a significant buffering effect against depressive symptoms (S. Liu et al., 2023). Second, depression and anxiety are two highly comorbid affective disorders, often coexisting as sister disorders (Kessler & Bromet, 2013). For psychological crisis, self-regulation and psychosomatic dysfunction had higher out-EI in the network compared with the other two dimensions, meaning that self-regulation and psychosomatic dysfunction can better predict psychological crisis among college students. Furthermore, self-regulation and psychosomatic dysfunction had strong correlations at both T1 and T2 with cognitive symptoms and somatic symptoms of anxiety, as well as affective disturbance and somatic disturbance of depression. The essence of these dimensions is the manifestation of an imbalance in the individual’s psychosomatic homeostasis system; thus, they can predict each other. In addition, psychological disturbance had a stronger in-EI than other dimensions, indicating that whether it is psychological crisis or anxiety, both are more likely to trigger psychological disturbance, a depressive symptom, in college students. When anxious, people pay more attention to negative information in life, even exaggerating its negative impact, which constitutes a negative cognitive bias (B. X. Liu & Li, 2018). When this effect persists and individuals cannot cope, it may be statistically associated with the psychological disturbance of learned helplessness (Wu et al., 2021). Moreover, positive cross-temporal predictive associations exist between nodes of psychological crisis and the psychological disturbance node of depression. This statistical association pattern suggests that there may be a mutually reinforcing relationship over time between psychological crisis and depression-related nodes, rather than a unidirectional causal chain. One possible explanation is that the cognitive and emotional dysregulation states accompanying psychological crisis may coexist empirically with negative cognitive bias; however, this interpretation awaits further empirical validation. It should be emphasized that, given that this study included only two measurement time points, the above predictive associations should be interpreted as exploratory findings, and future research should employ tracking designs with more time points to examine their potential directionality and stability. Second, according to the adaptation theory perspective, the psychological symptoms accompanying depression, such as long-term helplessness and negative self-evaluation, significantly weaken an individual’s psychological resilience (L. Han & Li, 2014), causing them to lack effective adaptation strategies when facing stressful events, thus making them more prone to falling into acute psychological crisis. Finally, from a systemic theory perspective, the individual and their environment, interpersonal relationships, and cultural background constitute a dynamic whole. Changes in any element of the system may cause imbalances in the individual’s cognition, emotion, or behavior, manifesting as anxiety or depressive symptoms. When an individual who is already in an imbalanced state encounters a stressful event, their psychological system is more likely to decompensate, thereby inducing a psychological crisis.

In summary, anxiety symptoms, depressive symptoms, and psychological crisis among college students do not exist in isolation but rather constitute a dynamic relationship network of mutual influence and interweaving, and this interaction shows a certain degree of stability over time.

### 4.4. Limitations and Future Directions

This study has several limitations. First, the sample was drawn from a specific region in China, which may affect the external validity and generalizability of the results. Future research should extend to more provinces, cities, and institutions to improve generalizability within China. Moreover, there may be cultural differences between Chinese and Western college students in the manifestations of psychological crisis (Lew et al., 2020). Future studies should, within a cross-cultural comparative framework, examine the moderating role of cultural factors (collectivism, individualism, family expectations, social evaluation pressure, etc.) in the symptom network. Second, we used questionnaire surveys, which may be affected by face validity, social desirability bias, and response set; future research should consider behavioral data to enhance the reliability of the results. Third, all college students involved in this study were from non-clinical samples, and their psychological crisis primarily manifested as situational and developmental psychological distress, rather than major mental disorders. Therefore, whether the findings of this study are applicable to college students who have been diagnosed with major mental disorders (schizophrenia, bipolar disorder, etc.) remains to be further verified. Future research could include clinical samples for comparison to examine the cross-group stability of the psychological crisis network structure. Finally, we did not organize psychological crisis, anxiety symptoms, and depressive symptoms into single variables but analyzed them by dimensions; some dimensions were not discussed in this study. In the future, the three could be organized into single variables, or only the influence of a certain dimension on other variables could be discussed to ensure a thorough analysis of the results.

## 5. Conclusions

Using longitudinal network analysis, in this study, we elucidated the intrinsic relationships among the dimensions of anxiety symptoms, depressive symptoms, and psychological crisis in college students. The main conclusions are as follows: (1) Anxiety, depression, and psychological crisis among college students each have core symptom nodes within their respective dimensions. The core symptom of anxiety was somatic symptoms at T1, which shifted to cognitive symptoms at T2; the core symptom of depression was affective disturbance at both time points; and the core symptom of psychological crisis was social support deficit and self-cognitive distress at both time points. These core symptoms demonstrated relative cross-temporal stability in the cross-sectional networks. (2) There is an internal connection mechanism among anxiety symptoms, depressive symptoms, and psychological crisis in college students. The connection between “social support deficit and self-cognitive distress” and “self-regulation and psychosomatic dysfunction” is the strongest in college students’ psychological crisis; the “motor symptoms-psychological disturbance” dimension shows the strongest connection between anxiety and depressive symptoms; and anxiety symptoms and depressive symptoms are correlated with psychological crisis. (3) The psychomotor disturbance dimension of depression has significant predictive power for each dimension of psychological crisis and anxiety symptoms in college students. (4) Self-regulation and psychosomatic dysfunction in college students’ psychological crisis are more strongly predicted by their anxiety and depressive symptoms. (5) All dimensions of psychological crisis and anxiety significantly predict the psychological disturbance dimension of depression.

## Data Availability

The datasets generated and analyzed during the current study are available from the corresponding author on reasonable request.

## Appendix A

**Table A1:** T1 Table of regularized partial correlation network edge coefficients.

	F1	F2	F3	A1	A2	A3	A4	B1	B2	B3	B4
**F1**	0.00	0.34	0.25	0.10	0.05	−0.03	0.00	0.06	0.04	0.07	0.04
**F2**	0.34	0.00	0.03	0.14	0.11	−0.19	0.01	0.14	0.04	0.11	0.00
**F3**	0.25	0.03	0.00	0.07	0.16	−0.05	0.00	0.00	0.04	0.03	0.00
**A1**	0.10	0.14	0.07	0.00	0.21	0.20	0.03	0.21	0.01	0.10	0.03
**A2**	0.05	0.11	0.16	0.21	0.00	0.02	0.13	0.09	0.25	0.10	0.00
**A3**	−0.03	−0.19	−0.05	0.20	0.02	0.00	0.19	0.00	−0.03	−0.03	0.30
**A4**	0.00	0.01	0.00	0.03	0.13	0.19	0.00	0.09	0.18	−0.01	0.16
**B1**	0.06	0.14	0.00	0.21	0.09	0.00	0.09	0.00	0.17	0.22	0.15
**B2**	0.04	0.04	0.04	0.01	0.25	−0.03	0.18	0.17	0.00	0.14	0.25
**B3**	0.07	0.11	0.03	0.10	0.10	−0.03	−0.01	0.22	0.14	0.00	−0.11
**B4**	0.04	0.00	0.00	0.03	0.00	0.30	0.16	0.15	0.25	−0.11	0.00

**Table A2:** Node strength values at T1 and T2.

	F1	F2	F3	A1	A2	A3	A4	B1	B2	B3	B4
**T1 strength**	0.97	1.104	0.61	1.09	1.098	1.04	0.80	1.14	1.15	0.93	1.03
**T2 strength**	0.95	1.13	0.65	1.12	1.11	1.00	0.83	1.19	1.15	0.89	1.06

**Table A3:** T2 Table of regularized partial correlation network edge coefficients.

	F1	F2	F3	A1	A2	A3	A4	B1	B2	B3	B4
**F1**	0.00	0.34	0.29	0.09	0.05	−0.01	0.00	0.04	0.03	0.05	0.05
**F2**	0.34	0.00	0.00	0.14	0.09	−0.21	0.03	0.18	0.06	0.07	0.00
**F3**	0.29	0.00	0.00	0.07	0.16	−0.05	0.00	−0.01	0.03	0.03	−0.01
**A1**	0.09	0.14	0.07	0.00	0.18	0.21	0.01	0.23	0.00	0.14	0.05
**A2**	0.05	0.09	0.16	0.18	0.00	0.03	0.15	0.08	0.27	0.09	0.00
**A3**	−0.01	−0.21	−0.05	0.21	0.03	0.00	0.18	−0.01	−0.02	0.00	0.27
**A4**	0.00	0.03	0.00	0.01	0.15	0.18	0.00	0.09	0.18	−0.03	0.16
**B1**	0.04	0.18	−0.01	0.23	0.08	−0.01	0.09	0.00	0.16	0.22	0.17
**B2**	0.03	0.06	0.03	0.00	0.27	−0.02	0.18	0.16	0.00	0.16	0.24
**B3**	0.05	0.07	0.03	0.14	0.09	0.00	−0.03	0.22	0.16	0.00	−0.11
**B4**	0.05	0.00	0.00	0.05	0.00	0.27	0.16	0.17	0.24	−0.11	0.00

**Table A4:** Expected influence values for each node at T1 and T2.

	F1	F2	F3	A1	A2	A3	A4	B1	B2	B3	B4
**T1 expected value**	0.91	0.72	0.52	1.09	1.10	0.37	0.78	1.13	1.09	0.63	0.81
**T2 expected value**	0.93	0.71	0.52	1.12	1.11	0.38	0.77	1.15	1.11	0.61	0.82

**Table A5:** Edge coefficient table for the cross-lagged network linking psychological crisis, anxiety, and depression.

	F1	F2	F3	A1	A2	A3	A4	B1	B2	B3	B4
**F1**	0.00	0.04	−0.01	0.04	0.03	0.00	0.01	0.01	0.03	0.01	0.08
**F2**	0.06	0.00	0.00	0.06	0.09	−0.02	0.02	0.07	0.06	0.01	0.09
**F3**	−0.04	−0.13	0.00	−0.05	−0.05	0.01	0.01	−0.02	−0.02	−0.01	0.00
**A1**	0.04	0.02	0.00	0.00	0.02	0.01	0.01	0.05	0.04	0.02	0.10
**A2**	0.00	0.02	0.00	0.03	0.00	0.02	0.02	0.02	0.08	0.01	0.03
**A3**	−0.01	−0.11	0.01	0.04	0.05	0.00	0.02	−0.02	−0.04	0.00	0.27
**A4**	0.02	−0.01	0.08	0.02	0.13	0.06	0.00	0.03	0.12	0.01	0.10
**B1**	0.04	0.16	0.02	0.09	0.05	−0.02	0.01	0.00	0.04	0.02	0.17
**B2**	0.04	0.08	0.01	0.05	0.13	0.01	0.05	0.05	0.00	0.01	0.06
**B3**	0.11	0.18	0.13	0.17	0.16	0.04	−0.04	0.01	0.00	0.00	0.06
**B4**	0.05	0.04	0.04	0.04	0.07	0.03	0.02	0.04	0.06	0.00	0.00

**Table A6:** Values of node strength, node expected influence, out-EI, and in-EI.

Note	Node Strength	Out-EI	In-EI	(Total) Node Expected Influence
**B4**	1.35	0.40	0.95	1.35
**A2**	1.00	0.21	0.68	0.89
**B3**	1.00	0.81	0.08	0.89
**B2**	0.97	0.49	0.37	0.86
**B1**	0.92	0.57	0.24	0.81
**A1**	0.88	0.32	0.48	0.80
**F2**	1.27	0.45	0.27	0.72
**A4**	0.78	0.55	0.12	0.67
**F1**	0.64	0.23	0.30	0.53
**A3**	0.78	0.21	0.15	0.36
**F3**	0.64	−0.31	0.29	0.60
